# Relationships between Molecular Structure of Carbohydrates and Their Dynamic Hydration Shells Revealed by Terahertz Time-Domain Spectroscopy

**DOI:** 10.3390/ijms222111969

**Published:** 2021-11-04

**Authors:** Nikita V. Penkov

**Affiliations:** Federal Research Center “Pushchino Scientific Center for Biological Research of the Russian Academy of Sciences”, Institute of Cell Biophysics RAS, 142290 Pushchino, Russia; nvpenkov@rambler.ru

**Keywords:** THz-TDS, hydration shells, carbohydrate hydration, water structure, dielectric properties, polysaccharides

## Abstract

Despite more than a century of research on the hydration of biomolecules, the hydration of carbohydrates is insufficiently studied. An approach to studying dynamic hydration shells of carbohydrates in aqueous solutions based on terahertz time-domain spectroscopy assay is developed in the current work. Monosaccharides (glucose, galactose, galacturonic acid) and polysaccharides (dextran, amylopectin, polygalacturonic acid) solutions were studied. The contribution of the dissolved carbohydrates was subtracted from the measured dielectric permittivities of aqueous solutions based on the corresponding effective medium models. The obtained dielectric permittivities of the water phase were used to calculate the parameters describing intermolecular relaxation and oscillatory processes in water. It is established that all of the analyzed carbohydrates lead to the increase of the binding degree of water. Hydration shells of monosaccharides are characterized by elevated numbers of hydrogen bonds and their mean energies compared to undisturbed water, as well as by elevated numbers and the lifetime of free water molecules. The axial orientation of the OH(4) group of sugar facilitates a wider distribution of hydrogen bond energies in hydration shells compared to equatorial orientation. The presence of the carboxylic group affects water structure significantly. The hydration of polysaccharides is less apparent than that of monosaccharides, and it depends on the type of glycosidic bonds.

## 1. Introduction

It is well known that hydration shells are an important factor determining the structure and function of biological molecules [1,2,3,4]. Carbohydrates, which are biological molecules without any doubt [5], are not an exception. The hydration of carbohydrates is of great importance for both basic and applied science. For example, many problems of taste chemistry [6,7], bioprotection [8,9], and food science [10] cannot be fully explained without taking the processes of carbohydrate hydration into account.

Studies of carbohydrate hydration shells started more than 100 years ago [11]. In the following years, more and more modern experimental methods capable of analyzing hydration were used, such as the measurement of vapour water pressure above an aqueous solution [12], densitometry [13], viscometry [14], measurement of water sorption [15], differential scanning calorimetry [16,17,18], X-ray diffraction method [19], compressibility measurement [20], ultrasonic techniques [21,22], NMR-spectroscopy [23,24], neutron scattering [25,26,27,28], extended depolarized light scattering [29,30], dynamic light scattering [31,32], IR-spectroscopy [33,34,35], Raman spectroscopy [27,36], polarization-resolved femtosecond-infrared spectroscopy [37], dielectric spectroscopy [38,39,40], terahertz (THz) spectroscopy [39,41,42,43,44,45,46,47], and molecular modeling [9,42,48,49,50,51,52]. However, despite such a rich history of studying carbohydrate hydration, we are still far from a complete understanding of this process. For example, there is no clear answer to a simple question: are the sugars cosmotropic or chaotropic agents [16,39,53]? Consequently, the studies by currently available methods and the development of novel approaches should be continued.

Each of the mentioned methods analyzes hydration using certain criteria, and, of course, the results obtained by different methods could be different. For example, calculations of the hydration number of sucrose molecules based on the data from different methods gave the following values: calorimetry—6.3 [54]; viscometry—11.2 [14]; acoustic methods—13.8 [14]; THz spectroscopy—35 [55].

THz spectroscopy reveals the highest number of water molecules in hydration shells compared to all the other experimental methods. This is due to the specifications of the THz spectral range corresponding to the intermolecular organization of water according to the specific times and energies. This is the only method sensitive to so-called dynamic hydration shells [42,45,56,57,58], which includes not just strongly bound water molecules in the primary layer, but also more distant molecules with slightly altered dynamics. In the case of aqueous sugar solutions, it is obviously confirmed by the fact that with increasing concentrations, the hydration number decreases starting from quite low concentrations, 0.15–0.3 M by monosaccharides [39,41,55]. This is explained by the overlapping of the distant outer layers of dynamic hydration shells of sugar molecules. Thus, THz spectroscopy is one of the most fruitful methods for studying the hydration of carbohydrates.

Among several modifications of the THz spectroscopy method, THz time-domain spectroscopy (THz-TDS) should be particularly mentioned. This method allows not just to measure the absorption spectrum, but also to determine the complex dielectric permittivity (DP), which contains significantly greater amounts of information on intermolecular structure and dynamics of the studied substance.

From the point of view of dielectric properties, the solution of carbohydrates is a heterogeneous biphasic system composed of water with inclusions of carbohydrate molecules, or triphasic, if regarding the phase of hydration shells separately. Such systems are usually considered in the dielectric physics using effective medium models [59,60,61], which allow taking into account the mutual polarization of phases and obtaining the correct relation of the DPs of heterogeneous systems with the DPs of the composing phases. It is worth noticing that one of the first works on studying aqueous sucrose solutions by THz-TDS method [47] gave rise to the question on the requirement for applying effective medium models, although the authors did not find the suitable solutions themselves. Despite that, the majority of the works in this field does either not use the effective medium models at all [16,37,39,41,43,62], or exploits a primitive additive model for the description of absorption spectra [42,44,45]. In the latter case, three of the aforementioned solution phases were taken into consideration, but the dielectric interaction between them was not taken into account. In other words, this approach is not related to the effective medium model. The only exception that we found in the literature is the paper [46]. The authors performed a deep analysis of the DPs of a glucose-water mixture in the whole concentration range from crystalline glucose to very diluted solutions. This allowed them to determine the ratio between different phases and to calculate the parameters of hydration shell water in the THz region. For this purpose, one of the well-known effective medium models, namely the Landau–Lifshitz–Looyenga model [63], was used, which is usually applied to heterogeneous systems with a low permittivity contrast between the phases.

The analysis of literature on studying aqueous carbohydrate solutions by THz spectroscopy allows to establish that all these studies cover only monosaccharides and small oligosaccharides. The studies of aqueous polysaccharide solutions are almost absent. At the same time, there is no reason to suppose that the characteristics of dynamic hydration shells of the monomers could be simply extrapolated to polymers. For example, one of our recent works [64] showed that the effect of DNA on water is significantly higher than the effect of equimolar nucleotide concentration.

**Figure 1:**
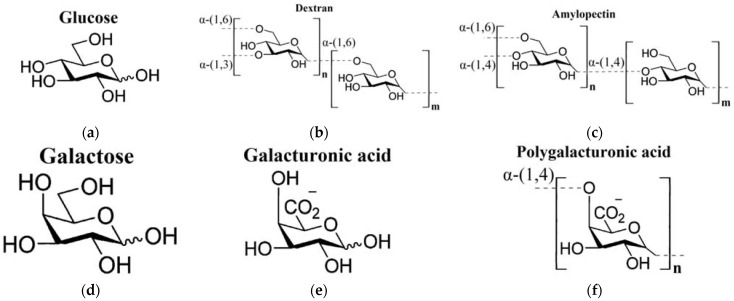
Chemical structures of the studied carbohydrates [66]: (**a**) glucose; (**b**) dextran and (**c**) amylopectin (polysaccharides of glucose); (**d**) galactose (a stereoisomer of glucose); (**e**) galacturonic acid; (**f**) polygalacturonic acid.

In the current work, the THz-TDS method was used to study aqueous polysaccharide solutions (dextran, amylopectin, polygalacturonic acid) compared to the aqueous solutions of their building monomers, or similar in chemical structure, monosaccharides (glucose, galactose, galacturonic acid). The structural formulae of all the analyzed carbohydrates are shown in Figure 1. When analyzing the DPs of carbohydrate solutions, we applied the corresponding effective medium models for calculation of the DPs of the water phase of the solutions. The Maxwell Garnett model was used for monosaccharides [60], whereas for polysaccharides, we used the effective medium model elaborated in our work [65].

## 2. Results

### 2.1. Characteristics of the Analyzed Carbohydrates in Dry Form

**Figure 2:**
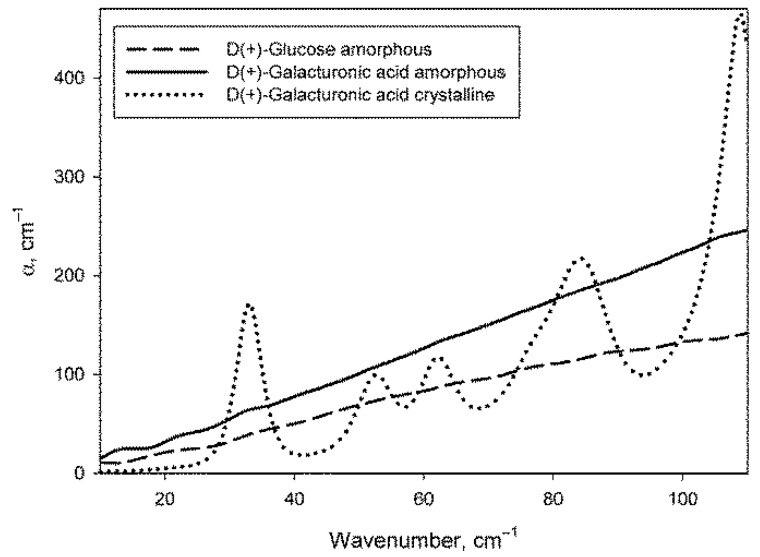
Absorption coefficient spectra of amorphous samples of glucose and galacturonic acid, as well as of crystalline galacturonic acid.

The procedure for preparing dry monosaccharides by melting them (see Section 4.3) allowed to obtain samples in an amorphous form, as evidenced by the absence of phonon crystal bands of monosaccharides in their spectra. Figure 2 shows the absorption spectra of glucose and galacturonic acid after melting (there are no phonon bands) in comparison with the spectrum of galacturonic acid without melting (with the obvious presence of phonon bands). The spectrum of amorphous galactose did not differ from the spectrum of amorphous glucose. The spectrum of crystalline glucose can be found in [67], and the spectrum of crystalline galactose is shown in paper [68].

**Figure 3:**
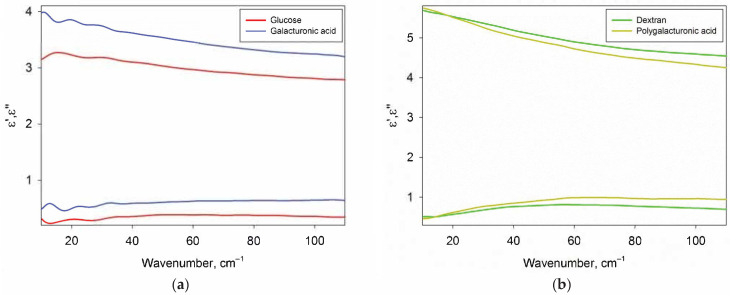
The real ε’ (top) and imaginary ε” (bottom) parts of the DPs of carbohydrates. Panel (**a**) contains DPs of amorphous monosaccharides (glucose and galacturonic acid), panel (**b**) contains DPs of polysaccharides (dextran and polygalacturonic acid).

Figure 3 shows the DPs of glucose, galacturonic acid, dextran and polygalacturonic acid in dry form. Glucose and galactose DPs were indistinguishable, so only glucose data are presented. In addition, the DPs of amylopectin practically did not differ from the DPs of dextran, therefore only dextran data are presented.

The DPs of carbohydrates were used to subtract the contribution of carbohydrates from the DPs of their aqueous solutions and to calculate the DPs of the water phase of the solutions (Section 2.3) The absence of phonon bands is of fundamental importance for the correct calculation, since the sugars in the solution are in a monomolecular form and also do not contain phonon bands.

The method of obtaining amorphous sugars used in this work turned out to be better than the method described in the paper [67], where the sugars were melted in an open container. Though the authors mentioned the short period of the melted state of the sugars, they nevertheless noticed their darkening. It could apparently be due to thermal decomposition of the sugars accompanied by dehydration reactions [69]. In this work, glucose and galactose samples were absolutely transparent after melting, whereas galacturonic acid displayed only a slightly yellowish colour. The applied approach of melting sugar in a thin layer between two surfaces prevents the evaporation of water formed during the decomposition of sugar. Since dehydration reactions during sugar decomposition are reversible [69], it is possible to preserve the chemical composition of the studied sugars.

### 2.2. Solutions Conductivity

The conductivity of the analyzed aqueous carbohydrate solutions was measured and used to calculate the parameters of the model DP (Section 4.6). Significant conductivity values were observed only in the solutions of carbohydrates susceptible to dissociation, namely galacturonic acid (1.3 S/m) and polygalacturonic acid (1.86 S/m). The conductivities of all other solutions were lower than 0.008 S/m and they were considered to be zero in the calculations.

### 2.3. DPs of Water Phase of Carbohydrate Solutions

**Figure 4:**
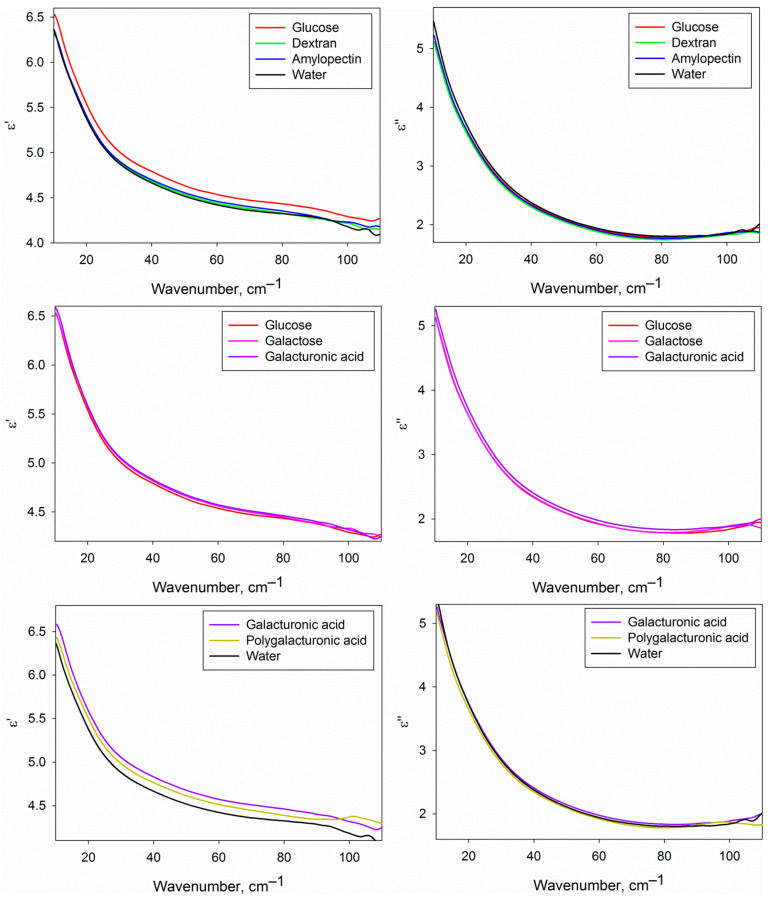
Dielectric permittivities (real $\varepsilon^{\prime }$ and imaginary $\varepsilon^{\prime \prime }$ parts) of the water phase of the studied carbohydrate solutions compared to each other and to water.

**Figure 5:**
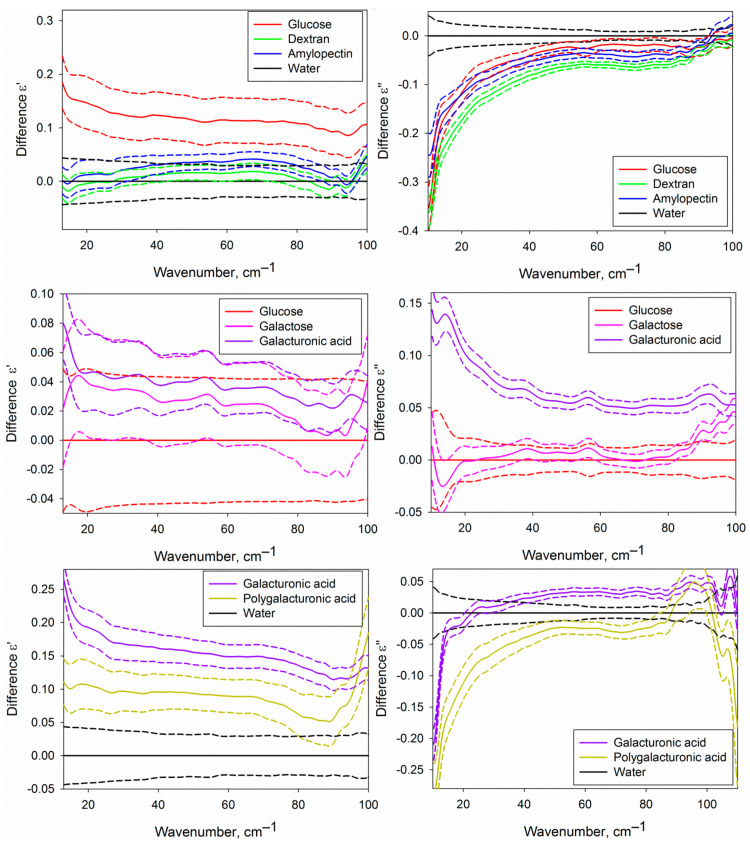
Differential dielectric permittivities (real $\varepsilon^{\prime }$ and imaginary $\varepsilon^{\prime \prime }$ parts) of the water phase of the studied aqueous carbohydrate solutions. The upper part corresponds to the solutions of glucose, dextran and amylopectin compared with water; the central part, to the solutions of galactose and galacturonic acid compared to glucose solution; lower part, to the solutions of galacturonic and polygalacturonic acid compared to water. Solid lines represent averaged DPs, and dashed lines of the same colour show the dispersion as a 95% mean confidence interval.

Figure 4 shows the real and imaginary parts of the DPs of the water phase of the studied carbohydrate solutions compared with each other and with pure water. We should note that the given DPs are related only to the water phase of the solutions, because the contribution of dissolved carbohydrates was excluded by effective medium models (Equations (2) and (3), see Section 4.5). DPs from Figure 4 provide basic knowledge of the dielectric characteristics of water in the analyzed solutions in the THz region, but the differential DPs shown in Figure 5 are more convenient to compare them with each other. 

Figure 5 shows certain differences of the DPs of the water phase of the solutions of different carbohydrates from pure water and from each other. It is conditioned by differences in the structural and dynamic characteristics of water in solutions caused by the formation of hydration shells of carbohydrates. The approach used in the present work does not allow to determine the DPs of hydration shells themselves, but since they comprise a fraction of the water phase volume, we can analyze them indirectly.

### 2.4. Parameters of the Model DPs of the Water Phase of Carbohydrate Solutions

As a rule, the THz spectra of aqueous solutions do not include sharp spectral bands (Figure 4), as it can be seen in mid IR or GHz ranges. This is the reason for the complexity and sometimes ambiguity of the interpretation of THz spectra of the liquids. To obtain data that could be clearly interpreted, the parameters of the model DP (Equation (5)) were calculated, which are presented in Table 1.

The parameters of Table 1 contain the integral characteristics of DPs and have a certain physical meaning. The analysis of these parameters of solutions compared to each other and to pure water allows interpreting the intermolecular structure and dynamics of water, which requires a detailed discussion.

## 3. Discussion

Aqueous carbohydrate solutions with concentrations of approximately 5% *w*/*w* were studied in the work. This concentration was regarded as optimal due to two reasons. Firstly, at concentrations below 3%, it was not possible to record reliable differences of the solutions from pure water. Secondly, it follows from the literature [39,41,55], that the ratio of hydration water volume to carbohydrate volume in the solutions of carbohydrates (mono- and disaccharides) decreases with increasing concentrations, starting from approximately 3–5%. A conclusion was made that it is related to partial overlapping of the hydration shells. Meanwhile, THz spectroscopy is of special value due to its ability to record the distant hydration shell layers [42,45,56,57,58]. Thus, the concentration below 5% made the method sensitivity insufficient, whereas the higher concentration would lead to an underreporting of data on the outer layers of the dynamic hydration shells.

The data from Table 1 show that, irrespective of the type of carbohydrate molecules in the solution, the hydration process is reflected in the decrease of the Δε_1_ parameter compared to pure water. This shows [70] a higher binding degree and lower mobility of water molecules included into the hydration shells of the carbohydrates. Such a conclusion is confirmed by other studies of sugar solutions by dielectric spectroscopy methods [38,71] and extended depolarized light scattering [29]. This can be explained by the hydrophilic nature of the studied carbohydrates, i.e., the binding of water to them is stronger than the binding of water molecules to each other [39,52]. Other parameters (except Δε_1_) demonstrate a wide diversity of the differences from water depending on the type of the dissolved carbohydrate.

Aqueous solutions of monosaccharides (glucose, galactose, galacturonic acid) demonstrate higher values of ω_0_, A/ω_0_^2^, Δε_2_, n and τ_2_ parameters compared to pure water (Table 1). Let us discuss each of the parameters separately.

A higher value of the frequency of intermolecular oscillations ω_0_ in monosaccharide solutions gives evidence on the elevation of the mean energy of hydrogen bonding. Hydrogen bonds of water with hydroxyl groups of sugars are stronger than with other water molecules [39,52], which explains the observed tendency.

Parameter A/ω_0_^2^ is a measure of the contribution of intermolecular oscillations into the overall dielectric response of the water phase (by analogy with Δε value for molecular relaxation). Higher values of this parameter means either a greater amplitude of hydrogen bond oscillations or a higher number of the hydrogen bonds. As it was mentioned above, increased energy of hydrogen bonds is observed in monosaccharide solutions, which is usually accompanied by reduced bond length and oscillation amplitude [72]. Thus, a conclusion was made about a greater number of hydrogen bonds in the hydration shells of monosaccharides. The analogous manifestation of hydration was observed in our recent works for Mg^2+^∙ATP complex [58] and DNA [64]. Apparently, it is a universal characteristic of hydration of a certain type of hydrophilic molecules. Let us clarify that the average number of hydrogen bonds for a single water molecule in pure water at 25 °C is equal to 3.6 of 4 theoretically possible [73]. Therefore, claiming an increased number of hydrogen bonds in hydrate shells implies an increase of no more than 10% compared to undisturbed water. It is a rather weak effect, but nevertheless, it could be revealed by the THz-TDS method.

The increase of parameters Δε_2_ and n in the presence of monosaccharides (Table 1) gives evidence of the presence of a higher number of free water molecules in their hydration shells compared to pure water [74,75]. Such manifestation of hydration for different classes of molecules was also discussed in the works [39,43,64,76,77]. As a rule, the presence of tightly bound water molecules in primary hydration shells is accompanied by the presence of a higher fraction of free water molecules in the outer hydration layers. This is explained by the fact that the rigid structure of water in the primary hydration shell differs significantly from the structure of pure water. As a result of such a difference of two water structures, a transient layer with a higher destruction degree and a higher number of free water molecules is observed between them. For example, microwave and THz spectroscopy used in the work [39] showed that disaccharide molecules in aqueous solution, beside binding to water molecules, facilitate the formation of 1.25 free water molecules per sugar molecule on average. The problem of determination of free water molecule content is quite complex, and different works exploit different approaches. Generally, the calculations of free water molecule fractions do not take screening processes into account. We addressed this issue in our work [78], as a result of which formula (7) was obtained. However, in this case, the correspondence of the tendencies of the fraction of free water molecules to increase upon hydration revealed in different works is important.

The increase of relaxation time of free water molecules τ_2_ (Table 1) is another specific feature of dynamic hydration shells of monosaccharides. We found this phenomenon during analysis of hydration of DPPC liposomes in a rippled gel phase [57], and protein molecules in a partially aggregated form [76], DNA [64]. In the mentioned works, an interpretation was made that we consider as applicable to general cases including monosaccharide solutions. Its essence is as follows. The structure of the hydration layer contains water molecules of different binding degrees, including a certain fraction of free molecules. The existence of water molecules in a free state is possible if it is confined in a certain cavity, and its walls composed of water molecules with fully occupied hydrogen bonding vacancies. Depending on the structure of such cavities, they affect orientational relaxation of free molecules more or less. Presumably, the structure of such cavities in hydration shells of monosaccharides is on average more compact than in pure water, which causes a retarding effect on free water relaxation (increases τ_2_). However, in this case, an approach based on molecular modeling should be determined to clarify this question completely.

When comparing the effect of three monosaccharides on water structure, galacturonic acid draws special attention. It is characterized by the highest values of Δε_1_, Δε_2_, τ_2_, γ, A/ω_0_^2^ parameters differing most significantly from pure water. Such manifestations are apparently due to the presence of a carboxylic group possessing a negative charge and extra hydrogen bonding site. To find out the relations between the mentioned parameters and the presence of carboxyl groups, additional studies are required involving a wider range of chemical compounds. In this case, we will just demonstrate the ability of the suggested approach to detect the difference of carbohydrates with different chemical groups based on the water dielectric properties in their solutions.

Differences between glucose and galactose solutions in γ parameter is even more interesting (Table 1). These two stereoisomers differ only in the orientation of the OH(4) group [79]. In glucose, it is oriented equatorially (Figure 1a), and in galactose, it is oriented axially (Figure 1d). All other differences (in α- to β-anomer ratio and content of furanose forms in aqueous solution) are insignificant [80]. Parameter γ has a meaning of width of the energy distribution of intermolecular hydrogen bonds, which is greater in the galactose solution. To understand this fact, the following works should be addressed, where NMR spectroscopy [23,24,81], compressibility measurements [79,82], and kinetic measurements [83] were used to establish the differences in water binding depending on the orientation of different OH-groups of sugars in aqueous solutions. It was shown that mutual orientation of the OH(4) and OH(2) groups is of the greatest importance. Monosaccharides with axial OH(4) and equatorial OH(2) groups, such as galactose and galacturonic acid, match the three-dimensional water structure, i.e., tetrahedral hydrogen bond network, much worse than monosaccharides with both equatorial groups, such as glucose. The worse they match, the greater the distortion of the water structure in the primary hydration shell. Obviously, this distortion is also transmitted to the more distant layers of the dynamic hydration shell. Apparently, it is accompanied by higher variability of intermolecular hydrogen bonding, which leads to wider energy distributions.

The crucial effect of the axial OH(4) group on the increase of γ parameter is confirmed as well in the case of the comparison of galacturonic and polygalacturonic acids. Galacturonic acid, as well as galactose, possess the axial OH(4) group (Figure 1e), and its solution is characterized by a higher γ value. Polygalacturonic acid has no OH(4) groups (Figure 1f) due to the presence of (1,4)-glycosidic bonds, and its solution is characterized by a lower γ value indistinguishable from glucose solution and water (Table 1).

Let us follow to comparing the characteristics of hydration of monomeric and polymeric forms of carbohydrates. It can be seen in Table 1 that aqueous solutions of polysaccharides (dextran and amylopectin) are characterized by a greater Δε_1_ value and lower values of three other parameters, Δε_2_, A/ω_0_^2^, n, compared to glucose. In addition, lower values of τ_2_ and ω_0_ values can be proposed for dextran and amylopectin, according to their mean values, although the differences are not statistically significant. In this case, polymerization leads to a shift of all the mentioned parameters to the side closer to the values of pure water. Moreover, the solutions of these polysaccharides become indistinguishable from water by all the parameters except Δε_1_.

The similar pattern is observed when comparing galacturonic and polygalacturonic acids. All the shown parameters of polygalacturonic acid solutions are closer to the values of water (Table 1). The difference of polygalacturonic acid from the glucose-based polysaccharides is that two of its parameters, ω_0_ and A/ω_0_^2^, are significantly higher than those of water, although lower than those of galacturonic acid. This is due to the carboxyl groups present in both monomers (Figure 1e) and polymer (Figure 1f). It contains two extra sites of rather strong hydrogen bonding. That is why oscillation frequency of the hydrogen bonds ω_0_ and their contribution to dielectric response determined by the A/ω_0_^2^ parameter are higher than in water.

Lower differences of aqueous polysaccharide solutions from water compared to monosaccharide solutions mean that the hydration shells of polysaccharides are less defined. This is not surprising because a part of the OH-groups of monosaccharides is transformed into glycosidic bonds upon polymerization. The hydration of glycoside oxygen is significantly lower than the hydration of the OH-group [48]. Moreover, each monosaccharide in the polymer is less available for water molecules due to the bonds with the adjacent monosaccharides. It is interesting to compare polysaccharides with nucleic acids in this regard. As we showed in the works [58,64], the same parameters of the model DP (Equation (5)) of the DNA solution differ from pure water parameters more than the parameters of nucleotide solutions of the corresponding concentrations. In other words, DNA affects water significantly stronger than separate nucleotides. This means that in the case of DNA we can speak of cooperative effects during the formation of hydration shells. This is in accordance with the results of our other work [57], where the estimation of the hydration layer of liposomes gave surprisingly high values, more than 5 nm, which could only be explained by cooperative effects. We do not observe such effects in aqueous polysaccharide solutions; on the contrary, we see the opposite tendency: polysaccharide induces less alterations in water structure than the composing monomers taken separately. It is possible that the manifestation of the cooperative effects of hydration require a significantly greater contact area of macromolecules with water compared to water molecule size. The diameter of liposomes is approximately hundreds of nanometers. The diameter of DNA molecules is approximately 2 nm, but its cross-section has a complex spatial structure with two grooves. The linear size of monosaccharides determining the cross-section of the polysaccharide chain is significantly smaller, approximately 0.7 nm [84].

It is interesting to compare amylopectin (Figure 1b) and dextran (Figure 1c). They are both branched homopolysaccharides composed of D-glucopyranose monomers. The ratio of branching points number to monomer number is similar in two polysaccharides. Their difference is in the glycosidic bonds linking the monosaccharides. In dextran [85,86], the main chain is linked by α-(1,6)-glycosidic bonds with α-(1,3)-branch linkages, whereas the main chain of amylopectin [87] contains α-(1,4)-glycosidic bonds and α-(1,6)-branch linkages. It can be seen in Table 1 that the solutions of dextran and amylopectin differ quite significantly by their Δε_1_ parameter values. On this basis, one can suggest that the approach proposed in this paper to the analysis of polysaccharide hydration is also sensitive to the type of glycosidic bonds. However, it is still unclear whether the main reason of the mentioned differences is the type of glycosidic bonds itself or the presence of OH-groups not included into glycosidic bonds, which require further studies on a wider range of substances.

Summarizing the obtained data, we can conclude that the THz-TDS method allows to obtain various information on the dynamic hydration shells of carbohydrates when using the described approaches to measurement, data transformation and analysis of spectra. This work also showed that the described characteristics of carbohydrate hydration in certain cases correlate clearly with their molecular structure. This means that the analysis of the hydration shells can give information about the structure of hydrated molecules. Of course, it is too early to talk about a full understanding of the relationship between the structure of these two substances, and extra studies are required, involving, for example, molecular modeling methods. However, it is already clear that the feasibility of THz-TDS as a structural method is not restricted to crystalline objects [67,88,89,90,91], but it could be developed to a consistent method of studying aqueous solution structures such as NMR and IR-spectroscopy. In addition, instead of the chemical shift and the position of spectral bands, THz spectroscopy should operate by the dielectric permittivity parameters of the water phase of the studied solutions.

## 4. Materials and Methods

### 4.1. Materials

The following carbohydrates were used in the work: D(+)-glucose anhydrous (#A1422, Panreac, Barcelona, Spain), D(+)-galactose (#A1131, Panreac, Spain), dextran ≈ 40 kDa (#31389, Sigma-Aldrich, Burlington, VT, USA), amylopectin (#10120, Sigma-Aldrich, USA), D(+)-galacturonic acid (#48280, Sigma-Aldrich, USA), polygalacturonic acid (#81325, Sigma-Aldrich, USA). The solutions were prepared using deionized water (MilliQ, Darmstadt, Germany). Sodium hydroxide (#567530, Sigma-Aldrich, USA) was used to elevate the pH of acidic solutions. Polyethylene powder (34–50 μm particle size, #434272, Sigma-Aldrich, USA) was used for preparation of the tablets with dry carbohydrates.

### 4.2. Solution Preparation

The carbohydrates solutions with the following concentrations were prepared: glucose and galactose, 50 mg/mL; galacturonic acid, 53.87 mg/mL; dextran and amylopectin, 45 mg/mL; polygalacturonic acid, 48.87 mg/mL. The reason for differences in the mass concentrations of monosaccharides is the difference in the molecular weight of glucose isomers (180.16 g/mol) and galacturonic acid (194.1 g/mol). Lower polysaccharide concentrations compared to monosaccharides are reasoned by the fact that in the composition of polysaccharides, monosaccharides are linked by glycosidic bonds, with the formation of each one accompanied by the cleavage of water molecules and a decrease of the molecular weight of the incorporated monosaccharide residue. Thus, the molar concentrations of all solutions for monosaccharides (277.5 mM) were equalized. It allowed the correct comparison of hydration shell parameters of different carbohydrates.

Dilution of monosaccharides in water was conducted by simple mixing and incubation for approximately 1 h at room temperature before the measurements. Polysaccharide solutions were prepared by the gradual addition of the dry substances to water at 80 °C with constant intense stirring by a magnetic stirrer. By the end of the polysaccharide dissolution (after achieving a visually uniform substance) the solutions were weighed and water lost due to evaporation during solution preparation was added.

The pH of the galacturonic and polygalacturonic acid solutions was adjusted to pH=6 by the addition of 1M NaOH solution.

### 4.3. Preparation of Dry Samples for Spectral Analysis

It is shown in Section 4.5, in addition to the spectra of carbohydrate solutions, the spectra of dry carbohydrates are required for the analysis of the characteristics of their hydration shells. Dry samples for spectral analysis were prepared by two different techniques.

The following procedure was adopted for the polysaccharides. 21 mg of each polysaccharide was mixed with 123 mg of polyethylene powder, after that the mixture was thoroughly ground in agate mortar. Then the powder was compressed at 10 tons to cylindrical pellet (13 mm in diameter and 1.1 mm in thickness). For background spectral measurement, a tablet of 123 mg pure polyethylene was prepared in an analogous manner.

Another technique was used for the monosaccharides. 30 mg of sugar was poured between two 5 µm Teflon films. The lower film was positioned on a smooth wooden surface for heat insulation. A flat metal heater with a temperature 3–5 degrees higher than the sugar melting point was applied to the upper film. After sugar melting, the heater was rapidly replaced by a metal plate cooled in liquid nitrogen. Due to the rather high glass transition temperature of sugars [92] and the thin layer of the sample, it was possible to cool it quickly and to obtain the monosaccharide in an amorphous phase.

Application of two different techniques of dry carbohydrate sample preparations for spectral analysis was related to the fact that the studied polysaccharides are amorphous, whereas monosaccharides are in a crystalline phase. The spectra of crystals display specific phonon absorption bands in the THz region. Since the spectra of dry samples were measured to subtract the contribution of carbohydrates from the DPs of their solutions (see Section 4.5), it was required to avoid the phonon oscillations because they are not present in the dissolved form of sugars. The use of monosaccharides in an amorphous form made it possible to address this issue.

### 4.4. THz-TDS

The THz-TDS assay is essentially the measurement of the time profile of the electric field of a picosecond pulse E(t). After the complex Fourier transformation of the E(t) of pulses transmitted through the sample and control without the sample (background pulse), the transmittance and refraction index spectra of the sample could be calculated. Based on these two spectra, the DP can be definitely calculated. The peculiarities of the THz-TDS method are well-known and they are described, for example, in [93]. The THz spectra were recorded on a TPS Spectra 3000 spectrometer (Teraview, Cambridge, UK) in a 10–110 cm^−1^ region with 4 cm^−1^ spectral resolution. For each spectrum, 1800 measured E(t) were averaged.

Spectral windows of liquid-phase cuvettes were made of z-cut quartz. All the solutions were degassed under 40 Torr vacuum for 15 min before the measurements using a degassing station (TA Instruments, New Castle, DE, USA). This prevented bubble formations on cuvette windows during spectral measurements. After placement of each sample into the sample compartment, a 10 min pause was held before the measurement. During this period, the sample temperature was stabilized at 25 ± 0.1 °C by a thermostatic holder, and the optic part was purged with dry air using an FT-IR Purge Gas Generator 74-5041 (Parker Hannifin Corporation, Haverhill, MA, USA).

A standard approach in the spectral analysis of liquid samples is the measurement of the sample spectrum in the cuvette and spectrum of empty cuvette as a background. However, such an approach leads to significant artifacts in the spectra [75]. To obtain the best quality spectra, we addressed this situation differently. Spectra of each sample were measured in two identical cuvettes differing only by inter-window distance (sample thickness). The spectrum of the solution in the thicker cuvette was regarded as «sample spectrum», and the spectrum of the same solution in the thinner cuvette was regarded as «background spectrum». The two mentioned spectra differ only in the thickness of the sample. As a result, a spectrum of solution with a thickness equal to the difference of thicknesses of two cuvettes was determined. Precise distances between the windows (50.06 and 100.26 µm) in two cuvettes were measured by recording the spectra of empty cuvettes in a FTIR spectrometer and further analysis of the etalon effect. Thus, we measured spectra of solutions with 50.2 µm thickness. The technique of measuring the spectra of solutions and determining the distances between windows is described in detail in [76].

DP was calculated from each pair of transmission Tr(υ) and refraction index n(υ) spectra of the solution by the following formulae [94]:

(1) $$\varepsilon^{\prime }\left(\upsilon \right)=n^{2}\left(\upsilon \right)-{\left[\frac{lnTr\left(\upsilon \right)}{4\pi \upsilon l}\right]}^{2},$$

$$\varepsilon^{\prime \prime }\left(\upsilon \right)=\frac{-n\left(\upsilon \right)lnTr\left(\upsilon \right)}{2\pi \upsilon l},$$

where $\varepsilon^{\prime }$ and $\varepsilon^{\prime \prime }$ are the real and imaginary parts of DP, υ is wavenumber, l is the thickness of the measured solution (50.2 µm in the current work).

DPs of dry carbohydrates prepared in forms of tablets or amorphous films (See Section 4.3) were also calculated by formulae (1), and their thicknesses were measured by a micrometer. The difference of thickness of sample-containing tablets and control tablets of pure polyethylene was taken as the thickness of polysaccharides in the sample.

To provide the possibility of statistical analysis, spectra of each sample were measured at least 20 times.

### 4.5. Calculation of DP of Water Phase of Carbohydrate Solution

The study of carbohydrate hydration was conducted on the basis of analysis of dielectric characteristics of water in their solutions. The carbohydrate solution can be regarded as a biphasic system from the point of view of dielectric properties, i.e., as a water phase with inclusions of carbohydrate molecules. To obtain the DP of the water phase, carbohydrate contribution must be subtracted from the measured DP of the solution. For this purpose, effective medium models establishing the relation between the DP of the biphasic system and DPs of both phases are used. Meanwhile, there is no effective medium model for the common case because its character depends on a number of parameters, for example, shape of the inclusions. The Maxwell Garnett model was used for monosaccharide solutions [60,95]:

(2) $$\frac{\varepsilon_{s}^{*}-\varepsilon_{w}^{*}}{\varepsilon_{s}^{*}+2\varepsilon_{w}^{*}}=f\frac{\varepsilon_{c}^{*}-\varepsilon_{w}^{*}}{\varepsilon_{c}^{*}+2\varepsilon_{w}^{*}},$$

where $\varepsilon_{s}^{*}$, $\varepsilon_{w}^{*}$, $\varepsilon_{c}^{*}$ are DPs of carbohydrate solution, its water phase and carbohydrate itself, respectively; *f* is the volume fraction of the carbohydrate in solution.

The Maxwell Garnett model is applicable for the biphasic systems with small spherical or chaotically oriented inclusions, which is true for monosaccharide solutions. For the polysaccharides, however, this model is not suitable, as well as all the other well-known models. In this regard, we developed an effective medium model for biphasic systems with fiber-like inclusions [65], which is applicable for the polysaccharide solutions. A simplified version of this model was used, applicable for low concentration solutions [65]:

(3) $$\varepsilon_{s}^{*}=\varepsilon_{w}^{*}+f\frac{\left(\varepsilon_{c}^{*}-\varepsilon_{w}^{*}\right)\left(5\varepsilon_{w}^{*}+\varepsilon_{c}^{*}\right)}{3\left(\varepsilon_{w}^{*}+\varepsilon_{c}^{*}\right)},$$

where the designations are the same as in formula (2).

Volume fractions *f* of the carbohydrates in solutions might be calculated by multiplying their mass concentrations (≈5%) and specific volumes of the carbohydrates. The specific volumes of the carbohydrates were taken from the literature: glucose and galactose, 0.62 cm^3^/g [82,84]; dextran, amylopectin, 0.6 cm^3^/g [96,97]; galacturonic and polygalacturonic acids, 0.55 cm^3^/g [82]. As it can be seen, the volume fraction of carbohydrates in all the solutions is approximately 3%, which makes the aforementioned approximation for low concentrations absolutely fair. Solution of the complex equation (3) makes it possible to find the real $\varepsilon_{w}^{\prime }$ and imaginary $\varepsilon_{w}^{\prime \prime }$ parts of $\varepsilon_{w}^{*}$:

$$\varepsilon_{w}^{\prime }=\frac{1}{e}\left(a-\sqrt{\frac{3}{2}\left(\sqrt{c^{2}+d^{2}}+c\right)}\right),$$

(4) $$\varepsilon_{w}^{\prime \prime }=\frac{1}{e}\left(b-\sqrt{\frac{3}{2}\left(\sqrt{c^{2}+d^{2}}-c\right)}\right),$$

$$a=\left(3+4f\right)\varepsilon_{c}^{\prime }-3\varepsilon_{s}^{\prime }$$

$$b=\left(3+4f\right)\varepsilon_{c}^{\prime \prime }-3\varepsilon_{s}^{\prime \prime }$$

$$c=3\left({\varepsilon_{s}^{\prime }}^{2}-{\varepsilon_{s}^{\prime \prime }}^{2}\right)+\left(6-28f\right)\varepsilon_{c}^{\prime }\varepsilon_{s}^{\prime }-2\left(3-14f\right)\varepsilon_{c}^{\prime \prime }\varepsilon_{s}^{\prime \prime }+\left(3+4f+12f^{2}\right)\left({\varepsilon_{c}^{\prime }}^{2}-{\varepsilon_{c}^{\prime \prime }}^{2}\right)$$

$$d=\left(6-28f\right)\varepsilon_{c}^{\prime \prime }\varepsilon_{s}^{\prime }+6\varepsilon_{s}^{\prime }\varepsilon_{s}^{\prime \prime }+\varepsilon_{c}^{\prime }\left(\left(6-28f\right)\varepsilon_{s}^{\prime \prime }+\left(6+8f+24f^{2}\right)\varepsilon_{c}^{\prime \prime }\right)$$

e=2(5f−3)

### 4.6. Analysis of Water Phase DP in Carbohydrate Solutions

To analyze the DP of the water phase of the solutions, we used a model DP described by the following equation:

(5) $$\varepsilon_{mod}^{*}=\frac{\Delta \varepsilon_{1}}{1-i\omega \tau_{1}}+\frac{\Delta \varepsilon_{2}}{1-i\omega \tau_{2}}+\frac{A}{\omega_{0}^{2}-\omega^{2}-i\omega \gamma }+\varepsilon_{\infty }+i\frac{\sigma_{0}}{\varepsilon_{0}\omega },$$

where $\tau_{1}$ and ${\Delta \varepsilon }_{1}$ are the time and strength of Debye relaxation of water [98,99]; $\tau_{2}$ and ${\Delta \varepsilon }_{2}$ and are the time and strength of relaxation of free or weakly bound water molecules [74,75,100]; A, $\omega_{0}$, γ are amplitude, resonance frequency and bandwidth parameter of intermolecular stretching oscillations of water molecules linked by hydrogen bonds [101,102]; $\varepsilon_{\infty }$ is high-frequency DP (a parameter describing the contribution of all the higher-frequency water polarization processes into the DP), $\sigma_{0}$ is a dc-conductivity of the solution, $\varepsilon_{0}$ is the electric constant, ω is the cyclic frequency, *i* is imaginary unit.

Model DP (Equation (5)) takes into account all the basic types of molecular dynamics of water displayed in the terahertz region. Separately, it can be noted that all the processes of molecular relaxation of carbohydrates in aqueous solution have specific times above 10 ps [40], and they are far beyond the THz region analyzed in this work. Therefore, in this paper they are excluded from consideration.

The model DP (Equation (5)) contains 9 parameters that could be calculated for each solution by fitting procedure. To reduce the fitting uncertainty, we reduced the number of variable parameters, as described further. Parameter $\sigma_{0}$ was measured separately (see Section 4.7). Parameter $\varepsilon_{\infty }$ was equated to 2.5; it is a specific value for diluted water solutions on frequencies around $\omega_{0}$. The maximum of the Debye relaxation band described by the first term of Equation (5) is approximately 0.6 cm^−1^ [103]. This is far from the analyzed frequency range (10–110 cm^−1^), where only the high-frequency part of the mentioned band is reflected. Upon water molecule binding, the amplitude of this band $\Delta \varepsilon_{1}$ decreases, whereas the relaxation time $\tau_{1}$ increases. Both these tendencies lead to reduced absorption in the low-frequency edge of the analyzed frequency region. In this regard, there is no need to treat both mentioned parameters independently. We fixed $\tau_{1}$ at an 8.28 ps value specific for pure water at 25 °C [103]. As a result, only 6 independent parameters were left out of 9 parameters of the model DP (Equation (5)), namely $\Delta \varepsilon_{1}$, $\Delta \varepsilon_{2}$, $\tau_{2}$, A, $\omega_{0}$, γ, which were calculated by fitting procedure.

The criterion of fitting was minimization of the *s* value:

(6) $$s=\frac{1}{N}\sqrt{\sum_{i=1}^{N}\left[{\left(\frac{\varepsilon_{mod}^{\prime }\left(\upsilon_{i}\right)-\varepsilon_{exp}^{\prime }\left(\upsilon_{i}\right)}{\varepsilon_{mod}^{\prime }\left(\upsilon_{i}\right)}\right)}^{2}+{\left(\frac{\varepsilon_{mod}^{\prime \prime }\left(\upsilon_{i}\right)-\varepsilon_{exp}^{\prime \prime }\left(\upsilon_{i}\right)}{\varepsilon_{mod}^{\prime \prime }\left(\upsilon_{i}\right)}\right)}^{2}\right]},$$

where «mod» and «exp» indices show model and experimental DPs of the water phase of solutions, N = 250 is a number of points in the spectrum. Fitting was performed for each pair of functions, $\varepsilon_{exp}^{\prime }\left(\upsilon \right)$ and $\varepsilon_{exp}^{\prime \prime }\left(\upsilon \right)$. The mean value of *s* comprised 0.001 and did not exceed 0.0021 for any of the separate calculations, which shows a high precision of fitting.

Using the calculated parameters of the model DP (Equation (5)), the share of free water molecules was calculated using the ratio obtained in our earlier work [78]:

(7) $$n=\frac{3\Delta \varepsilon_{2}}{\left(\Delta \varepsilon_{2}+\varepsilon_{\infty }+\frac{A_{1}}{\omega_{1}^{2}}+2\right)\left(\varepsilon_{\infty }+\frac{A_{1}}{\omega_{1}^{2}}+2\right)}*\frac{9kT\varepsilon_{0}}{Np^{2}}$$

where *k*—Boltzmann constant (1.38 × 10^−23^ J/K), $\varepsilon_{0}$—the electric constant, *p*—electric dipole moment of a water molecule (6.17 × 10^−30^ C·m [104]), $N=N_{A}\times 55.56\times 10^{3}$ molecules/m^3^—numerical concentration of water molecules, *N_A_*—Avogadro constant (6.02 × 10^23^ molecules/mol), *T*—solution temperature (298.15 K).

### 4.7. Measurement of Solution Conductivity

The measurement of conductivity $\sigma_{0}$ included into Equation (5) was carried out with high accuracy on Zetasizer nano ZS (Malvern, Malvern, UK) in zeta potential measurement mode. The measurements were carried out in capillary cuvettes with electrodes DTS1060 (Malvern, UK) at 25 °C.

## 5. Conclusions

An approach based on the THz-TDS assay was suggested in the current work for studying the dynamic hydration shells of the carbohydrates in aqueous solutions. Complex dielectric permittivities of the water phase were calculated from recorded spectra of carbohydrate solutions in the THz area using the corresponding effective medium models. Decomposition of these dielectric permittivities into terms related to relaxation of bound and free water molecules, as well as to intermolecular oscillations, allows to obtain the set of parameters with a clear physical sense in terms of molecular structure and dynamics of water. The obtained data give evidence that the binding degree of water is elevated in the hydration shells of all the studied carbohydrates compared to pure water. In addition, the hydration shells of monosaccharides are characterized by elevated numbers and average energy of hydrogen bonds, as well as by elevated numbers and relaxation time of free water molecules. In the hydration shells of sugars with an axially oriented OH(4) group, the distribution of intermolecular hydrogen bond energies is wider than in the case of equatorial orientation. The presence of a carboxylic group significantly affects the characteristics of the hydration shells. Polysaccharide hydration is significantly weaker than that of monosaccharides, and it depends on the type of glycosidic bonds.

## Figures and Tables

**Table 1:** Parameters of model DP (Equation (5)) calculated for all the studied aqueous carbohydrate solutions and pure water. Percentage of free water molecules n calculated by formula (7) is also given in the last column.

Carbohydrate	Δε_1_	Δε_2_	τ_2_, ps	ω_0_, cm^−1^	γ, cm^−1^	A/ω_0_^2^	n, %
Pure water	68.86 ± 0.81	2.691 ± 0.042	0.316 ± 0.006	207.2 ± 4.3	196.5 ± 11.1	1.702 ± 0.019	3.78 ± 0.04
Glucose	62.45 ± 0.48	2.939 ± 0.039	0.326 ± 0.005	215.9 ± 3.9	202.6 ± 5.6	1.807 ± 0.038	3.91 ± 0.03
Galactose	62.23 ± 0.83	2.931 ± 0.046	0.327 ± 0.004	221.3 ± 4.9	217.3 ± 9.0	1.844 ± 0.029	3.86 ± 0.04
Galacturonic acid	63.20 ± 0.46	3.006 ± 0.024	0.334 ± 0.003	221.7 ± 4.7	223.1 ± 9.6	1.867 ± 0.022	3.91 ± 0.03
Dextran	63.81 ± 0.88	2.728 ± 0.052	0.321 ± 0.005	210.6 ± 4.2	200.0 ± 10.0	1.719 ± 0.018	3.80 ± 0.05
Amylopectin	65.97 ± 0.84	2.688 ± 0.045	0.319 ± 0.005	209.7 ± 4.5	197.4 ± 9.6	1.737 ± 0.016	3.74 ± 0.04
Polygalacturonic acid	63.75 ± 0.84	2.729 ± 0.051	0.312 ± 0.004	219.1 ± 7.0	208.2 ± 13.0	1.801 ± 0.025	3.72 ± 0.06

The shown dispersions are presented as 95% mean confidence intervals.

## Data Availability

The data presented in this study are available on request from the corresponding author.
